# New Zealand blackcurrant extract enhances fat oxidation during prolonged cycling in endurance-trained females

**DOI:** 10.1007/s00421-018-3858-3

**Published:** 2018-04-04

**Authors:** Juliette A. Strauss, Mark E. T. Willems, Sam O. Shepherd

**Affiliations:** 1 Research Institute for Sport and Exercise Sciences, Liverpool John Moores University, Byrom Street, Liverpool, L3 3AF UK; 2 Institute of Sport, University of Chichester, Chichester, UK

**Keywords:** Anthocyanins, New Zealand blackcurrant, Polyphenols, Cycling, Substrate oxidation

## Abstract

**Purpose:**

New Zealand blackcurrant (NZBC) extract has previously been shown to increase fat oxidation during prolonged exercise, but this observation is limited to males. We examined whether NZBC intake also increases fat oxidation during prolonged exercise in females, and whether this was related to greater concentrations of circulating fatty acids.

**Methods:**

In a randomised, crossover, double-blind design, 16 endurance-trained females (age: 28 ± 8 years, BMI: 21.3 ± 2.1 kg·m^−2^, VO_2max_: 43.7 ± 1.1 ml·kg^−1^·min^−1^) ingested 600 mg·day^−1^ NZBC extract (CurraNZ^™^) or placebo (600 mg·day^−1^ microcrystalline cellulose) for 7 days. On day 7, participants performed 120 min cycling at 65% VO_2max_, using online expired air sampling with blood samples collected at baseline and at 15 min intervals throughout exercise for analysis of glucose, NEFA and glycerol.

**Results:**

NZBC extract increased mean fat oxidation by 27% during 120 min moderate-intensity cycling compared to placebo (*P* = 0.042), and mean carbohydrate oxidation tended to be lower (*P* = 0.063). Pre-exercise, plasma NEFA (*P* = 0.034) and glycerol (*P* = 0.051) concentrations were greater following NZBC intake, although there was no difference between conditions in the exercise-induced increase in plasma NEFA and glycerol concentrations (*P* > 0.05). Mean fat oxidation during exercise was moderately associated with pre-exercise plasma NEFA concentrations (*r* = 0.45, *P* = 0.016).

**Conclusions:**

Intake of NZBC extract for 7 days elevated resting concentrations of plasma NEFA and glycerol, indicative of higher lipolytic rates, and this may underpin the observed increase in fat oxidation during prolonged cycling in endurance-trained females.

## Introduction

Blackcurrant (*Ribes nigrum*) is one of the richest sources of polyphenols, and includes high concentrations of the anthocyanins delphinidin-3-rutinoside, delphinidin-3-glucoside, cyanidin-3-rutinoside, and cyanidin-3-glucoside. Anthocyanins are a major flavonoid subclass, and recent epidemiological studies demonstrate that higher anthocyanin intakes are related to lower arterial stiffness, blood pressure and risk of type 2 diabetes (Jennings et al. 2012; Wedick et al. 2012). These health benefits are thought to be mediated by the effect of anthocyanins on inflammatory responses, antioxidant activity and endothelial function (Liu et al. 2016; Pojer et al. 2013; Wallace et al. 2016). Moreover, blackcurrant intake increases forearm blood flow at rest (Matsumoto et al. 2005), potentially mediated by anthocyanin-induced vasodilation and vasorelaxation (Ziberna et al. 2013).

Recent studies have revealed a potential ergogenic effect of New Zealand blackcurrant (NZBC) extract intake on physiological and metabolic exercise responses and performance outcomes. Specifically, 7 days of NZBC intake (~ 105 mg anthocyanins per day) improved intermittent running (Perkins et al. 2015) and 16.1 km cycling time trial performance (Cook et al. 2015), and fat oxidation during 10 min cycling at ~ 65% VO_2max_ was 27% higher compared to placebo (Cook et al. 2015). More recently, Cook et al. (2017) demonstrated a dose–response effect of NZBC extract on fat oxidation during 2 h cycling at ~ 65% VO_2max_, with fat oxidation being 22 and 24% greater following 7 days supplementation with 600 and 900 mg NZBC, respectively (~ 210 and ~ 315 mg anthocyanins per day). Whilst demonstrating a clear benefit of short-term NZBC intake on fat oxidation and exercise performance, these studies were only conducted in male participants with no analysis of blood measures related to metabolic function. Therefore, studies are now required to determine if an ergogenic effect of NZBC intake on fat oxidation is also apparent in other populations.

When matched for age, BMI and fitness, females have higher body fat levels compared to their male counterparts, and exhibit lower rates of whole-body carbohydrate oxidation and greater rates of fat oxidation during exercise (Devries 2016). Less of a reliance on liver glycogen and possibly also reduced muscle glycogen utilisation during exercise underpins the lower rates of carbohydrate oxidation in females compared to males (Devries et al. 2006; Friedlander et al. 1998). Conversely, the rate of glycerol appearance in the blood is also elevated in females compared to males (Carter et al. 2001), indicative of greater lipolytic rates, although the source (plasma free fatty acids or intramuscular triglycerides) of glycerol remains contentious. Despite intramuscular triglyceride levels being greater in females (Devries et al. 2007; Tarnopolsky et al. 2007), studies investigating intramuscular triglyceride utilisation during exercise are equivocal, with some reporting greater (Roepstorff et al. 2002; Steffensen et al. 2002), less (Zehnder et al. 2005), or equal (Devries et al. 2007; White et al. 2003) utilisation when comparing males and females. Similarly, some studies report higher rates of free fatty acid and glycerol appearance during exercise in females compared to males (Davis et al. 2000; Mittendorfer et al. 2002), reflecting a greater capacity for adipose tissue lipolysis, although others report no differences (Romijn et al. 2000). Despite these discrepant findings, it is clear, however, that in the post-absorptive state there is a greater reliance on fat as a fuel source during exercise in females, and this is predominantly driven by the higher circulating oestrogen concentrations observed in pre-menopausal women (Devries 2016). Therefore, determining whether NZBC can further augment fat oxidation during exercise in females is now of interest.

Compared to males, the use of ergogenic aids to enhance fat oxidation during exercise in females has received comparatively less attention in the literature. The aim of this study was, therefore, to investigate whether short-term supplementation of NZBC extract could enhance fat oxidation in endurance-trained females during prolonged moderate-intensity exercise. We also measured plasma glucose, non-esterified fatty acids (NEFA) and glycerol at rest and throughout exercise to begin to investigate the potential mechanisms underpinning changes in substrate utilisation induced by NZBC extract.

## Method

### Subjects

16 healthy, active females (see Table 1 for subject characteristics) volunteered to take part in the study, which was approved by the Liverpool John Moores University Research Ethics Committee. Written, informed consent was obtained from volunteers following a verbal and written explanation of the nature and risks involved in the experimental procedures. Participant’s had a history of endurance sports participation of greater than 3 years, typically performing 5–10 h endurance-type exercise each week, of which at least two hours was cycling exercise.

**Table 1:** Participant characteristics (*n* = 16)

OC/NOC	7/9
Age (y)	28 ± 8
Height (m)	1.67 ± 0.06
Body mass (kg)	59.5 ± 8.4
BMI (kg m^−2^)	21.3 ± 2.1
VO_2max_ (L min^−1^)	2.63 ± 0.46
VO_2max_ (mL kg^−1^ min^−1^)	43.7 ± 1.1
VO_2max_ (mL kg FFM^−1^ min^−1^)	62.5 ± 7.1
W_max_ (W)	263 ± 45
HR_max_ (bpm)	188 ± 8
Lactate_peak_ (mmol L^−1^)	10.9 ± 1.9
Workload at 65% VO_2max_	125 ± 4
Daily anthocyanin intake (mg)	67 ± 14

Values are means ± SD

*BMI* body mass index, *HR*_*max*_ heart rate maximum, *NOC* not using oral contraceptive, *OC * oral contraceptive, *W*_*max*_ maximum workload

### Experimental design

Participants visited the laboratory on three separate occasions having abstained from vigorous exercise for 48 h and alcohol and caffeine for 24 h prior. On the first occasion, participant’s height and weight were measured, and body composition was assessed using electrical bioimpedance (Tanita BC 418 MA Segmental Body Composition Analyzer, Tanita, Japan). Initially, participant’s completed a submaximal graded-intensity exercise test on an electronically braked cycle ergometer (Lode BV, Groningen, The Netherlands), starting at 50 W and increasing by 30 W every 4 min, until a blood lactate ≥ 4 mmol L^−1^ was reached. After 15 min recovery, participants completed a progressive test to exhaustion on the same cycle ergometer to determine maximal oxygen uptake (VO_2max_) using an online gas collection system (Moxus Metabolic System, AEI Technologies, Pittsburgh, PA, USA). Briefly, participants cycled at 50 W for 4 min, after which the workload was increased by 30 W every 1 min until a cadence of ≥ 50 rpm could not be maintained. VO_2max_ was achieved when the following end-point criteria were met: (1) heart rate within 10 b min^−1^ of age-predicted maximum, (2) respiratory exchange ratio > 1.1, and (3) plateau of oxygen consumption despite increased workload (Gilman 1996).

In a randomised, double-blind, crossover design, participant’s then ingested 2 capsules (600 mg) of concentrated NZBC extract or a visually identical placebo for 7 days. This dose has previously been shown to lead to a ~ 22% increase in fat oxidation during 120 min of cycling at 65% VO_2max_ in endurance-trained males (Cook et al. 2017). Each 300 mg NZBC capsule contained 105 mg of anthocyanins, consisting of 35–50% delphinidin-3-rutinoside, 5–20% delphinidin-3-glucoside, 30–45% cyanidin-3-rutinoside, and 3–10% cyanidin-3-glucoside (CurraNZ^™^, Health Currancy Ltd, Surrey, UK). Each placebo capsule contained 300 mg microcrystalline cellulose. One capsule was consumed with breakfast and one with dinner (approximately 12 h apart) for the first 6 days. Seven participants were randomised to receive the NZBC supplement for their first trial. On the final morning of the supplementation period, participants arrived at the laboratory following an overnight fast (> 10 h) and first consumed a standardised breakfast providing 1 g.kg body mass^−1^ carbohydrate (typically consisting of porridge with semi-skimmed milk, orange juice and a cereal bar) and the final two capsules. 2 h following the standardised breakfast, participants completed a 120 min bout of steady-state exercise on an electronically braked cycle ergometer at a workload equivalent to ~ 65% VO_2max_. At rest and at 15 min intervals throughout the exercise bout, blood samples were collected from an indwelling cannula placed in the forearm of an antecubital vein, and expired air was collected using an online gas collection system (Moxus Metabolic System, AEI Technologies, Pittsburgh, PA, USA). Participants were provided with ad libitum access to water, and all exercise was conducted in a temperature controlled laboratory (19 °C). All experimental trials took place on day 9–11 of the follicular phase of the menstrual cycle, and therefore, the washout period between trials was ~ 28 days. In a previous study, an anthocyanin intake for 1 month at a dose greater than that used in the present study required a 15 day washout period for biomarkers of antioxidant status to return to baseline (Alvarez-Suarez et al. 2014).

### Habitual dietary intake and anthocyanin consumption

Dietary intake was recorded in a written diary for 48 h prior to the first experimental trial, and participants were instructed to replicate this before the subsequent trial (using the first diet diary as a guide). Food diaries were analysed using Nutritics software (Nutritics Ltd, Dublin, Ireland) for carbohydrate, protein and fat intake and total energy consumption (Table 2).

**Table 2:** Absolute and relative macronutrient and energy intake 48 h prior to each experimental trial

	NZBC	Placebo
Carbohydrate		
g	245 ± 67	253 ± 56
g kg body mass^−1^	4.1 ± 1.3	4.3 ± 1.5
Protein		
g	75 ± 21	70 ± 15
g kg body mass^−1^	1.3 ± 0.5	1.2 ± 0.5
Fat		
g	62 ± 11	71 ± 14
g kg body mass^−1^	1.0 ± 0.3	1.2 ± 0.4
Total energy intake		
kJ	7623 ± 1632	7724 ± 1795
kJ kg body mass^−1^	128 ± 30	130 ± 29

Values are means ± SD

At the first visit, participants also completed a food frequency questionnaire that listed the quantity and frequency of anthocyanin-containing foods and drinks compiled from the Phenol Explorer database (Neveu et al. 2010). By multiplying the anthocyanin content of the portion size by the total consumption frequency of each food, daily anthocyanin intake was calculated.

### Blood sample analysis

Plasma samples for each time point were obtained through centrifugation (10 min at 1000 g at 4 °C) and stored at − 80 °C for subsequent analysis. Plasma glucose, non-esterified fatty acids (NEFA) and glycerol concentrations were determined spectrophotometrically using a semi-automatic analyser in combination with commercially available kits (Randox Laboratories, Antrim, UK). Each sample was analysed in duplicate.

### Calculations and statistical analysis

Rates of whole-body fat and carbohydrate oxidation (g.min^−1^) were calculated from VO_2_ and VCO_2_ values collected during the steady-state cycling exercise, and were made assuming protein oxidation to be negligible, according to previously published equations (Jeukendrup and Wallis 2005):

$${\text{Carbohydrate oxidation }}\left( {{\text{g}}~{\text{mi}}{{\text{n}}^{ - {\text{1}}}}} \right)\,=\,{\text{4}}.{\text{21}}0\cdot{\text{VC}}{{\text{O}}_{\text{2}}}-{\text{2}}.{\text{962}}\cdot{\text{V}}{{\text{O}}_{\text{2}}}$$

$${\text{Fat oxidation}}\,=\,{\text{1}}.{\text{695}}\cdot{\text{V}}{{\text{O}}_{\text{2}}}-{\text{1}}.{\text{7}}0{\text{1}}\cdot{\text{VC}}{{\text{O}}_{\text{2}}}.$$

All data are expressed as means ± SD. Significance was set at the 0.05 level of confidence. Interpretation of 0.05 > *P* ≤ 0.1 was according to guidelines by Curran-Everett and Benos (2004). Time-dependent changes in substrate utilisation and blood metabolite concentrations during steady-state cycling exercise were compared between trials using a within-subjects repeated measures ANOVA. Significant main effects or interactions were assessed using Bonferroni adjustment *post hoc* analysis. All other data was compared using a paired students *t* test.

## Results

### Physiological data, energy expenditure and substrate oxidation

**Fig. 1:**
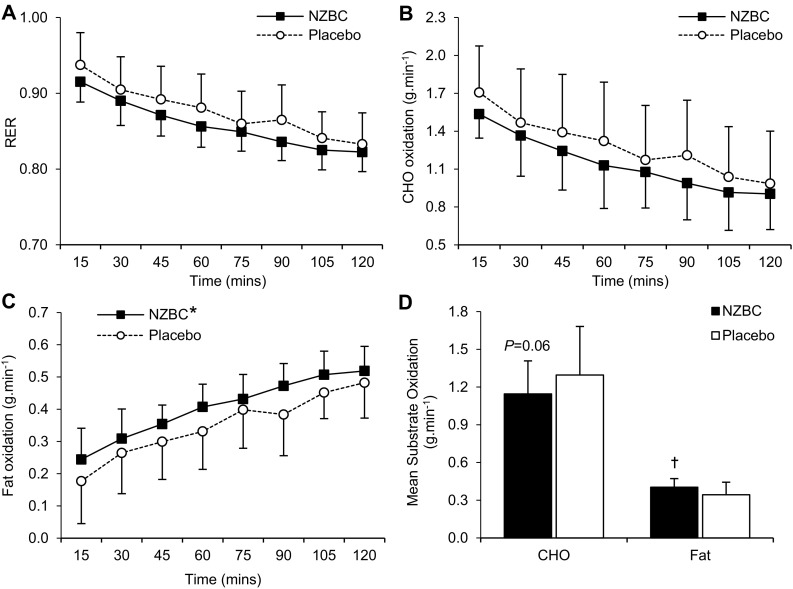
Respiratory exchange ratio (RER) (**a**), carbohydrate oxidation (**b**), fat oxidation (**c**), and mean rates of substrate oxidation (**d**) during 2 h cycling at ~ 65% VO_2max_ following 7 days supplementation with NZBC extract or placebo. Values are presented as mean ± SD. There was a main time effect for RER, carbohydrate and fat oxidation during the exercise bout (*P* < 0.001). *Main condition effect (*P* = 0.042). ^†^Significantly different from placebo (*P* = 0.047)

RER decreased over time during the steady-state cycling bout (*P* < 0.001; Fig. 1a), with a trend for RER to be lower in response to NZBC (main condition effect; *P* = 0.058). Accordingly, mean RER during the cycling bout tended to be lower in response to NZBC compared to placebo (*P* = 0.063). Carbohydrate oxidation decreased over time during the cycling bout (*P* < 0.001), and this tended to be different between NZBC and placebo (main condition effect; *P* = 0.063; Fig. 1b). The mean rate of carbohydrate oxidation also tended to be 12% lower in response to NZBC compared to placebo (*P* = 0.064; Fig. 1d). Fat oxidation increased over time during the cycling bout (*P* < 0.001), and was significantly greater during the NZBC trial (main condition effect; *P* = 0.042; Fig. 1c). As such, the mean rate of fat oxidation during the 2 h cycling bout was 27% higher following NZBC compared to placebo supplementation (*P* = 0.047; Fig. 1d). During the cycling bout, the relative contribution of carbohydrate and fat to total energy expenditure was decreased and increased, respectively (main time effect; *P* < 0.001). There also tended to be a condition effect (*P* = 0.059), such that relative carbohydrate oxidation was 11% lower (NZBC: 54 ± 7% vs. placebo: 63 ± 7%) and fat oxidation was 19% higher (NZBC: 46 ± 12% vs. placebo: 37 ± 12%), following NZBC compared to placebo.

During the 2 h steady-state cycling exercise there were no time or condition effects for heart rate, VO_2_, mean relative intensity, or energy expenditure. In contrast, there was a time effect for VCO_2_ (*P* = 0.002), with no difference between conditions (Table 3).

**Table 3:** Physiological data and energy expenditure during 2 h cycling following NZBC extract or placebo intake for 7 days

Condition	Time (min)							
	15	30	45	60	75	90	105	120
VO_2_ (L min^−1^)								
NZBC	1.74 ± 0.22	1.73 ± 0.25	1.72 ± 0.25	1.73 ± 0.28	1.74 ± 0.27	1.75 ± 0.27	1.76 ± 0.28	1.77 ± 0.29
Placebo	1.74 ± 0.24	1.72 ± 0.27	1.73 ± 0.28	1.74 ± 0.31	1.75 ± 0.29	1.75 ± 0.29	1.75 ± 0.31	1.77 ± 0.31
VCO_2_ (L.min^−1^)*								
NZBC	1.59 ± 0.19	1.54 ± 0.24	1.50 ± 0.24	1.49 ± 0.27	1.48 ± 0.25	1.47 ± 0.25	1.46 ± 0.26	1.46 ± 0.26
Placebo	1.63 ± 0.23	1.56 ± 0.27	1.55 ± 0.29	1.54 ± 0.31	1.51 ± 0.28	1.52 ± 0.29	1.48 ± 0.31	1.48 ± 0.30
% VO_2max_								
NZBC	66.7 ± 7.4	66.1 ± 6.6	65.7 ± 5.1	66.2 ± 6.0	66.7 ± 6.7	66.9 ± 5.8	66.4 ± 4.4	67.6 ± 4.4
Placebo	66.7 ± 7.3	65.8 ± 7.4	66.1 ± 7.1	66.2 ± 7.7	66.9 ± 7.0	66.8 ± 7.1	66.0 ± 5.7	66.8 ± 6.6
Heart rate (b min^−1^)								
NZBC	152 ± 17	153 ± 16	153 ± 16	153 ± 17	154 ± 15	154 ± 16	154 ± 16	156 ± 16
Placebo	153 ± 17	154 ± 17	155 ± 16	155 ± 16	156 ± 16	157 ± 15	158 ± 16	159 ± 15
Energy expenditure (kJ min^−1^)								
NZBC	35.3 ± 4.4	34.9 ± 5.2	34.6 ± 5.2	34.8 ± 5.8	34.8 ± 5.6	34.9 ± 5.5	35.0 ± 5.7	35.3 ± 5.8
Placebo	35.6 ± 4.8	34.9 ± 5.7	35.0 ± 5.8	35.0 ± 6.4	35.2 ± 5.9	35.2 ± 6.1	35.0 ± 6.4	35.3 ± 6.5

Values are means ± SD

*Main effect of time (*P* = 0.002)

### Blood parameters

**Fig. 2:**
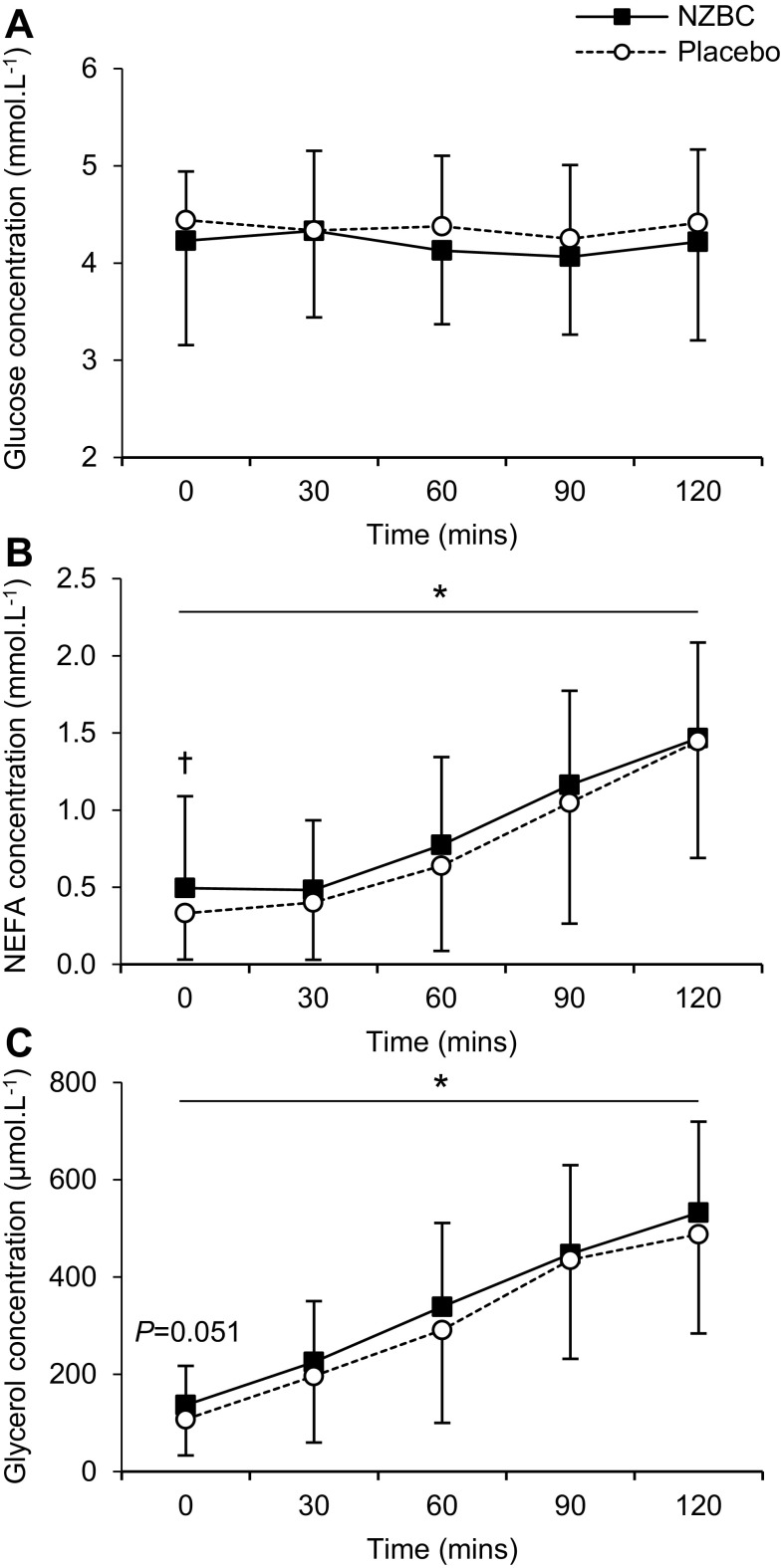
Plasma glucose (**a**), NEFA (**b**), and glycerol (**c**) concentrations during 2 h cycling at ~ 65% VO_2max_ following 7 days supplementation with NZBC extract or placebo. Values are presented as mean ± S.D. *Main time effect (*P* < 0.001). ^†^Significantly different from placebo at the equivalent time point (*P* = 0.034)

Pre-exercise plasma glucose concentrations were not different between conditions (*P* > 0.05; Fig. 2a). Pre-exercise plasma NEFA and glycerol concentrations were 49% (*P* = 0.034; Fig. 2b) and 27% (*P* = 0.051; Fig. 2c) higher, respectively, following NZBC supplementation compared to placebo. During the cycling bout, plasma NEFA and glycerol concentrations increased over time (*P* < 0.001; Fig. 2b, c), with no difference between conditions (*P* = 0.324). No time or condition effect was observed for plasma glucose (Fig. 2a). Pre-exercise plasma NEFA concentrations were moderately associated with mean rates of fat oxidation during exercise (*r* = 0.45, *P* = 0.016).

## Discussion

The novel findings from this study are that supplementation with NZBC extract for 7 days in endurance-trained females (1) enhanced fat oxidation during 120 min moderate-intensity cycling, and (2) increased pre-exercise plasma NEFA and glycerol concentrations. The latter observation suggests an effect of short-term NZBC intake on rates of lipolysis at rest, and thereby highlights one potential mechanism by which NZBC intake can enhance fat oxidation during exercise in females.

Evidence for nutritional supplements to increase fat oxidation during exercise is predominantly derived from studies conducted in males, and therefore, the effects of these supplements in females is largely unknown. Recently, it has been reported in two studies that NZBC intake for 7 days augmented whole-body fat oxidation during cycling at 65% VO_2max_ in endurance-trained males (Cook et al. 2015, 2017), but whether a similar effect is apparent in females has not been investigated. We now report for the first time that intake of NZBC extract for 7 days increased whole-body fat oxidation during prolonged moderate-intensity cycling in endurance-trained females. Moreover, fat oxidation was 27% higher with NZBC intake compared to the placebo condition, which is higher than the ~ 21.5% increase in fat oxidation reported by Cook et al. (2017) using the same exercise protocol and NZBC extract dose (600 mg day^−1^, containing 210 mg anthocyanins). Although a direct comparison between males and females in the same study is yet to be made, our data suggest that short-term intake of NZBC extract is at least as potent for increasing whole-body fat oxidation during exercise in females as previously observed in males.

The second novel finding of the present study was that 7 days NZBC intake increased pre-exercise plasma NEFA and glycerol concentrations. In addition, and as expected, plasma NEFA and glycerol concentrations increased throughout the prolonged exercise bout, but there was no difference between the two conditions. Together, these data indicate that NZBC extract increased adipose tissue lipolysis under resting conditions, and that plasma NEFA and glycerol were maintained at a higher concentration during exercise as a result. Moreover, pre-exercise plasma NEFA concentrations were moderately associated with fat oxidation, suggesting that the increase in lipolysis under resting conditions is an important determinant of the rate of fat oxidation during exercise. The precise mechanism by which NZBC extract enhances lipolysis is unknown, but could be related to the effect of NZBC anthocyanins or their metabolites on key proteins regulating lipolysis. For example, treating adipocytes isolated from rats with the anthocyanin cyanidin-3-glucoside for 24 h augments mRNA expression of the key lipolytic enzyme hormone-sensitive lipase (HSL) and the lipid droplet protein, perilipin 1 (Tsuda et al. 2005). NZBC extract contains high levels of cyanidin-3-glucoside, and therefore, 7 days NZBC intake may have increased HSL and perilipin 1 expression in adipose tissue leading to greater rates of lipolysis, although these responses are speculative and warrant further examination. Ultimately, though, an increase in the rate of lipolysis would increase the availability of plasma FFA available to be taken up into skeletal muscle and oxidised as a substrate during exercise.

An increase in lipolysis is only one possible mechanism by which NZBC may have enhanced fat oxidation. For example, blackcurrant ingestion increased peripheral blood flow during a maximal voluntary contraction of the trapezius muscle following typing activity (Matsumoto et al. 2005), which could subsequently enhance delivery of fatty acids to skeletal muscle. Anthocyanin intake could also have direct effects on skeletal muscle. For example, AMP-activated protein kinase (AMPK) protein expression and phosphorylation is elevated in skeletal muscle of mice following 5 weeks ingestion of an anthocyanin-rich bilberry extract (Takikawa et al. 2010). AMPK activation is important, because it can induce translocation of the primary fatty acid transporter in skeletal muscle, FAT/CD36, to the plasma membrane and, therefore, increase fatty acid uptake (Luiken et al. 2003). Furthermore, AMPK inhibits the activity of acetyl-CoA carboxylase thereby suppressing malonyl-CoA production and increasing fatty acid entry into the mitochondria (Towler and Hardie 2007). It is possible, therefore, that increased fat oxidation following anthocyanin intake can be realised through the effects of anthocyanins on several nodes of control related to protein activity and expression in adipose tissue and skeletal muscle.

The increased fat oxidation following NZBC intake in our female participants appears to be valid, since the mean difference of ~ 27% in fat oxidation is greater than the 10% day-to-day variability in fat oxidation reported previously (Achten and Jeukendrup 2003), and is greater than the reported variation in fat oxidation of 3 to 6% during exercise lasting more than 1 h (Hodgson et al. 2013). We should also note that blood samples were obtained 3 h postprandial of breakfast (that aimed to provide 1 g kg^−1^ carbohydrate), and, therefore, cannot be classified as representing the fasted state per se. However, pre-exercise blood glucose concentrations were ~ 4.3 mmol L^−1^, which is similar to or even lower than blood glucose concentrations typically observed following an overnight fast, and were not different between conditions. This is important, because it indicates that insulin concentrations were also likely to be low in both conditions, and the suppressive effect of insulin on adipose tissue lipolysis would be minimal.

We employed a 7 day supplementation period in this study as previous studies used this strategy to show increased fat oxidation during exercise in male participants (Cook et al. 2015, 2017). However, from this approach it is not possible to determine whether the increase in fat oxidation is reflective of an acute or chronic supplementation effect. Anthocyanin bioavailability is relatively poor, with only ~ 12% of anthocyanins appearing in the blood following ingestion (Czank et al. 2013), but anthocyanin metabolites remain in the blood up to 48 h following intake (Kay et al. 2005). Therefore, the intake of NZBC for 7 days will likely lead to an accumulation of anthocyanin metabolites over time which subsequently resulted in the increase in fat oxidation.

The habitual intake of anthocyanins was calculated to be 67 ± 14 mg day^−1^ using a food frequency questionnaire, and is, therefore, in agreement with previously published estimates of flavanol intake (including anthocyanins) of 51 mg day^−1^ in males (Zamora-Ros et al. 2011). This highlights that the daily dose of anthocyanins provided by NZBC extract (210 mg) for 7 days was much larger than the dose present in the habitual diet of our participants. We also did not find a relationship between habitual anthocyanin intake and fat oxidation during exercise, suggesting that anthocyanin intake from dietary sources alone is insufficient to impact substrate utilisation. Moreover, Cook et al. (2017) reported that a dose of 105 mg day^−1^ was insufficient to significantly enhance fat oxidation during exercise in endurance-trained males, whereas in the same study, fat oxidation was increased using a dose of 210 mg day^−1^ anthocyanins. Therefore, the dose of NZBC required to substantially enhance fat oxidation during exercise in both male and female participants is likely to be much greater than can be achieved through ingesting unprocessed anthocyanin-rich foods alone.

In summary, we show for the first time that 7 day NZBC intake augments fat oxidation during 120 min moderate-intensity exercise in endurance-trained females. Furthermore, we show that NZBC intake increases resting plasma NEFA and glycerol concentrations, thereby highlighting a potential mechanism by which NZBC increases fat oxidation.

## Abbreviations

AMPK: AMP-activated protein kinase
FAT/CD36: Fatty acid translocase/cluster of differentiation 36
HSL: Hormone-sensitive lipase
NEFA: Non-esterified fatty acids
VO_2max_: Maximum oxygen uptake
